# Supplementary material for: Bcl-xL targeting eliminates ageing tumor-promoting neutrophils and inhibits lung tumor growth
Source: EMBO Mol Med. 2023 Dec 20;16(1):10. doi: 10.1038/s44321-023-00013-x (PMC10897164; doi:10.1038/s44321-023-00013-x)
Supplement: Supplementary file 1 — Appendix [file 44321_2023_13_MOESM1_ESM.pdf]

Appendix

Bcl-xL targeting eliminates ageing tumor-promoting neutrophils and inhibits lung tumor growth

Table of Contents

APPENDIX FIGURE S1 – GATING STRATEGY USED FOR FLOW CYTOMETRY 2

APPENDIX FIGURE S2 – BCL-XL BLOCKADE DOES NOT SENSITIZE KP TUMORS TO ANTI-PD1 3

APPENDIX TABLE S1 – PATIENT CHARACTERISTICS AND CLINICAL DATA 4

APPENDIX TABLE S2 – ANTIBODIES USED FOR FLOW CYTOMETRY 5

APPENDIX TABLE S3 – DATASETS USED FOR HUMAN TRANSCRIPTOMICS ANALYSES 6

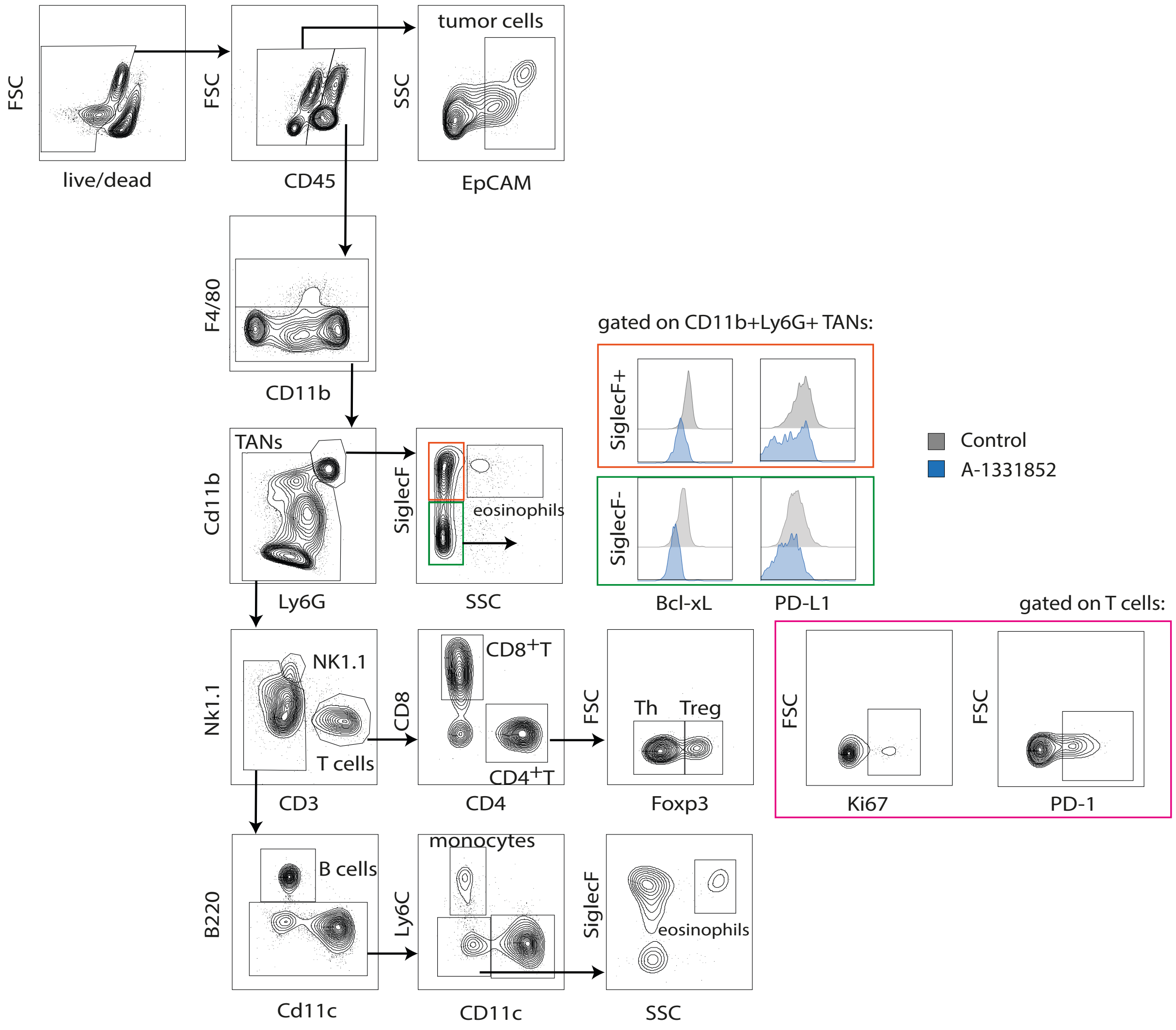

Appendix Figure S1 – Gating strategy used for flow cytometry.
Data were analyzed using FlowJo. The selection of cells was done based on their size and granularity (FSC-A versus SSC-A), followed by doublets and dead-cell exclusion. The following cell populations were analyzed based on the indicated gating strategy: tumor cells (CD45- EpCAM+), macrophages (CD45+ F4/80+), neutrophils (CD45+ F4/80- Cd11b+ Ly6G+ SCC^{low}), SiglecF+ neutrophils (CD45+ F4/80- Cd11b+ Ly6G+ SCC^{low} SiglecF+), NK cells (CD45+ F4/80- Cd11b+ Ly6G- NK1.1+), CD8 T cells (CD45+ F4/80- Ly6G- NK1.1- CD3+, CD8 +), CD4 T cells (CD45+ F4/80- Ly6G- NK1.1- CD3+, CD4 +), Tregs (CD45+ F4/80- Ly6G- NK1.1- CD3+, CD4 + Foxp3+), B cells (CD45+ F4/80- Ly6G- NK1.1- CD3- B220+), monocytes (CD45+ F4/80- Ly6G- NK1.1- CD3- B220- Ly6C+), dendritic cells (CD45+ F4/80- Ly6G- NK1.1- CD3- B220- Ly6C- Cd11c+) and eosinophils (CD45+ F4/80- Ly6G- NK1.1- CD3- B220- Ly6C- Cd11c- SiglecF+ SCC^{high}). Ki67 and PD-1 expression was further assessed in the different T cell populations.

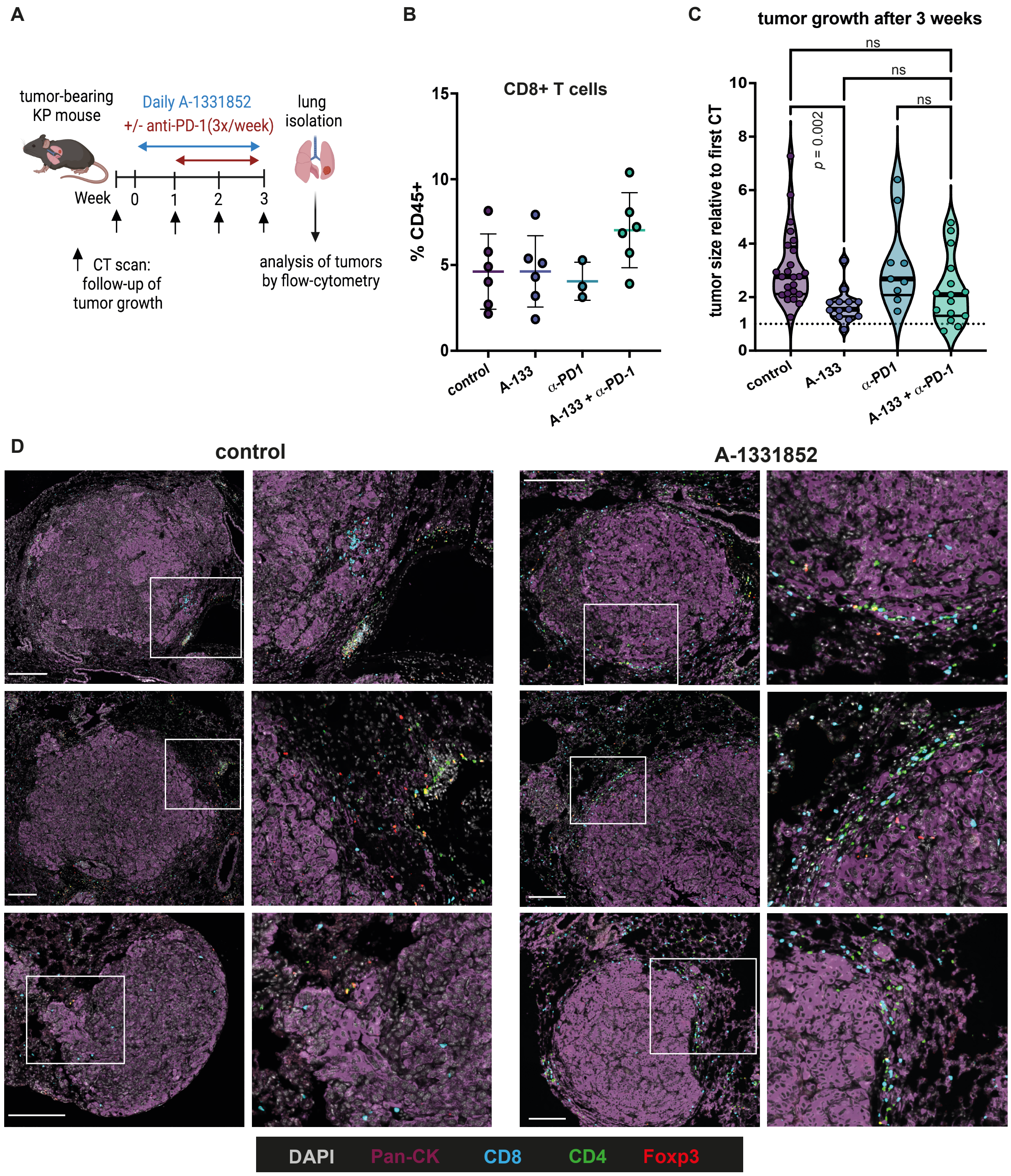

Appendix Figure S2 – Bcl-xL blockade does not sensitize KP tumors to anti-PD1.

(A) Scheme showing the experimental design. Tumor-bearing mice were treated with A-1331852 (n=3) for three weeks, with or without anti-PD-1 (n=3) during the last two weeks. **(B)** Percentage of CD8+ T cells among CD45+ cells in tumors of control (n=6), A-1331852 (A-133, n=6), anti-PD1 (n=3) or A-1331852 + anti-PD1 (n=6). **(C)** Tumor growth relative to the first tumor volume measured and after three weeks. Data are shown as mean ± S.D. Each data point represents a single tumor analyzed. For **(C)**, significance was determined by the Kruskal-Wallis test followed by Dunn's multiple comparisons test. ns, non-significant. **(D)** Representative examples and magnified views of multiplex-immunofluorescence of tumors from control or A-1331852-treated mice. Scale bars: 200 μm.

Sample ID	Date of diagnosis	Date of surgery	Sex/Age at diagnosis	Primary tumor - WHO grade/TNM stage/Morphology/Localisation	Driver gene mutation/fusion	Time of recurrence (weeks)	Treatment	Response date
20B17143	10/2020	23/11/2020	F/73	1 / pT1cN0 / M-81403 / C34.1	p53, ROS1		Radiation therapy - 11/2021	01/2022
20B18379	11/2020	14/12/2020	F/73	2 / pT2N0 / M-81403 / C34.3	KRAS, p53		Pharmacotherapy - 29/01/2021-30/04/2021 (4 cures)	06/2021
19B21488	11/2019	25/11/2019	F/59	1 / pT1cNxMx / M-81403 / C34.3	Not done	20	Pharmacotherapy - 2 cures	
20B08210	12/2019	22/06/2020	M/49	2 / pT4N0Mx / M-81403 / C34.1	p53	4	Pharmacotherapy - 03/2020 - 11/2020 (4 cures) Radiation therapy - 01/2021	
20B10321	06/2020	27/07/2020	M/69	2 / pT1cN0Mx / M-81403 / C34.1	Not done			
21B10918	04/2020	21/06/2021	F/62	1 / pT1bN0 / M-81403 / C34.2	EGFR			
21B02484	12/2020	08/02/2021	M/52	2 / pT2N2Mx / M-81403 / C34.1	p53, BRAF		Pharmacotherapy - 17/03/2021 - 04/06/2021 (4 cures)	08/2021

Appendix Table S1 – Patient characteristics and clinical data.
Clinical data of LUAD patients from which tumor sections were used for immunofluorescence staining in Fig. 6B.

Antibody	Source	Reference (Cat#)	Dilution
Bcl-xL-PE	Abcam	ab208747	1/500
SiglecF-PeVio 615	Miltenyi BioTec	130-112-172	1/100
CD45-Pacific orange	ThermoFisher scientific	MCD4530	1/250
CD45-PerCP	Miltenyi BioTec	130-102-785	1/250
Ly6G-PE	BioLegend	127607	1/400
Ly6G-PerCP-Cy5.5	BioLegend	127615	1/400
Ly6C-PerCP	BioLegend	128027	1/250
CD11b-BV711	BioLegend	101242	1/250
CD11c-BV421	BioLegend	117330	1/250
F4/80-BV605	BioLegend	123133	1/200
CD3-PE-Cy5.5	eBioscience	35-0031-82	1/250
CD4-AF700	BioLegend	100536	1/250
CD8a-BV570	BioLegend	100740	1/250
FoxP3-PE-eFluor 610	eBioscience	61-5773-82	1/100
B220-PE-Cy5	BioLegend	103209	1/250
Nk1.1-BV650	BioLegend	108736	1/250
PD-1-PE-Cy7	BioLegend	329917	1/200
PD-L1-BV785	BioLegend	124331	1/200
EpCAM-APC	eBioscience	15-5791-82	1/200
Ki67-FITC	BD Pharmingen	556026	1/50
BrdU-APC	BioLegend	339808	1/10

Appendix Table S2 – Antibodies used for flow cytometry.

	Number of samples used (total patients)	Age (median, range)	Gender	Stage	Dataset Name	Institution	Source	Reference
Lung-0 Dataset	82 (82)	61 (35-79)	F (36) / M (46)	I (56) / II (26)	DFCI	Dana-Farber Cancer Institute	Director's Challenge Consortium	Nat Med 14, 822–827 (2008)
	79 (79)	68 (36-87)	F (39) / M (40)	I (41) / II (20) / 3 (15) / NA (3)	HLM	Moffitt Cancer Center	Director's Challenge Consortium	Nat Med 14, 822–827 (2008)
	28 (90)	63 (38-75)	F (13) / M (15)	NA (28)	JBR	Ontario Cancer Institute	Zhu *et al.*	J Clin Oncol. 2010 Oct 10; 28(29): 4417–4424
	177 (178)	65 (33-86)	F (77) / M (100)	I (116) / II (29) / III (32)	MI	University of Michigan	Director's Challenge Consortium	Nat Med 14, 822–827 (2008)
	104 (104)	65 (38-82)	F (67) / M (37)	I (63) / II (20) / III (21)	MSKCC	Memorial Sloan-Kettering Cancer Center	Director's Challenge Consortium	Nat Med 14, 822–827 (2008)
Lung-0 Dataset (total)	470 (533)	65 (33-87)	F (232) / M (238)	I (276) / II (95) / III (68) / NA (31)				

Appendix Table S3 – Datasets used for human transcriptomics analyses.
Datasets used for *CSF2* expression analyses in Fig. EV2F.
