# Supplementary figures and images for: Bcl-xL targeting eliminates ageing tumor-promoting neutrophils and inhibits lung tumor growth
Source: EMBO Mol Med. 2023 Dec 20;16(1):10. doi: 10.1038/s44321-023-00013-x (PMC10897164; doi:10.1038/s44321-023-00013-x)

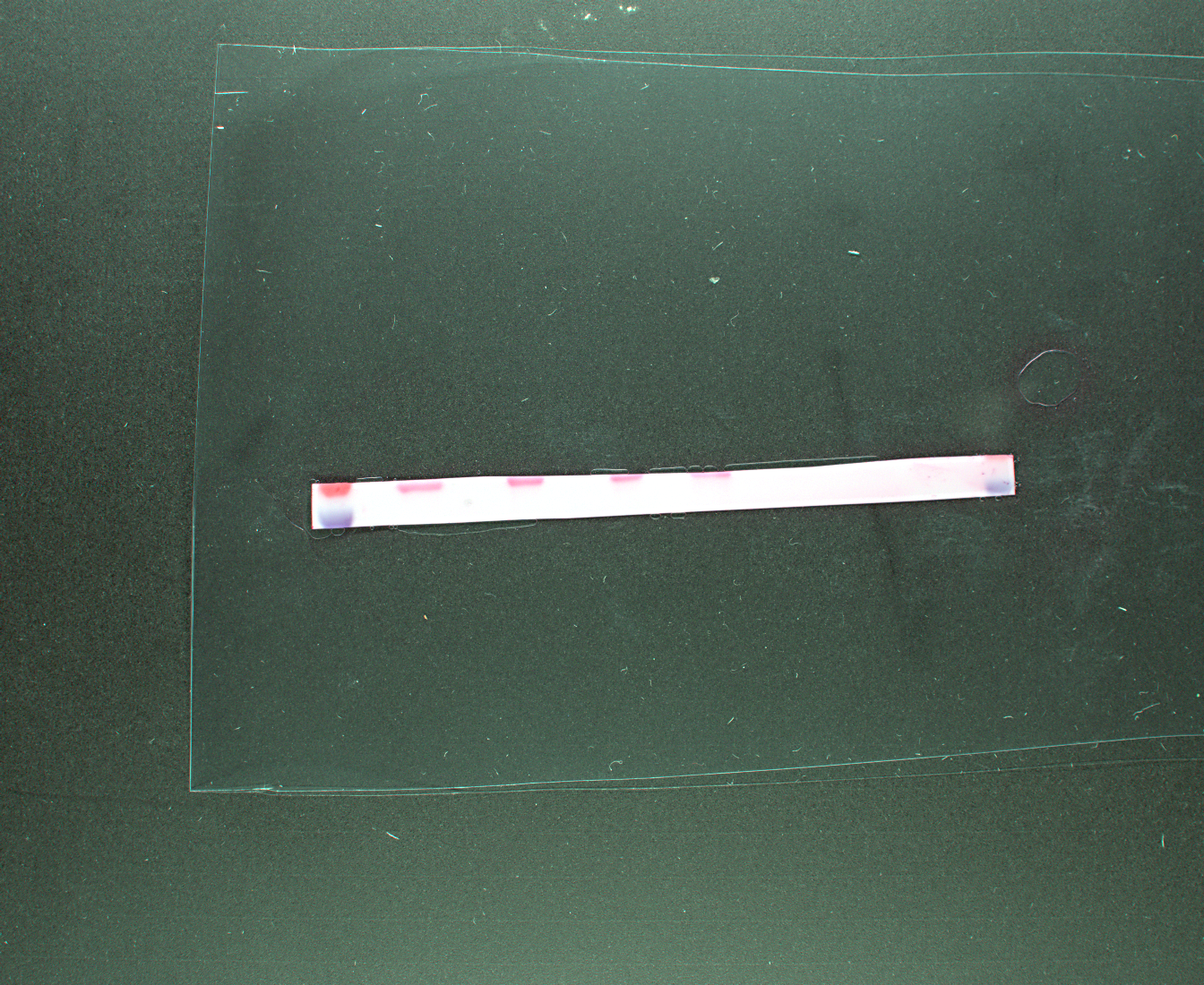

Supplement: Supplementary file 3 — Source Data Fig. 1 [file 44321_2023_13_MOESM3_ESM.zip › EMM-2023-18237_SourceDataForFigure1/1H/western Ponceau.tiff]

medium      SV2 SN      med  
+ A-133      SV2 SN  
+ A-133

35 kDa

25 kDa

50 kDa

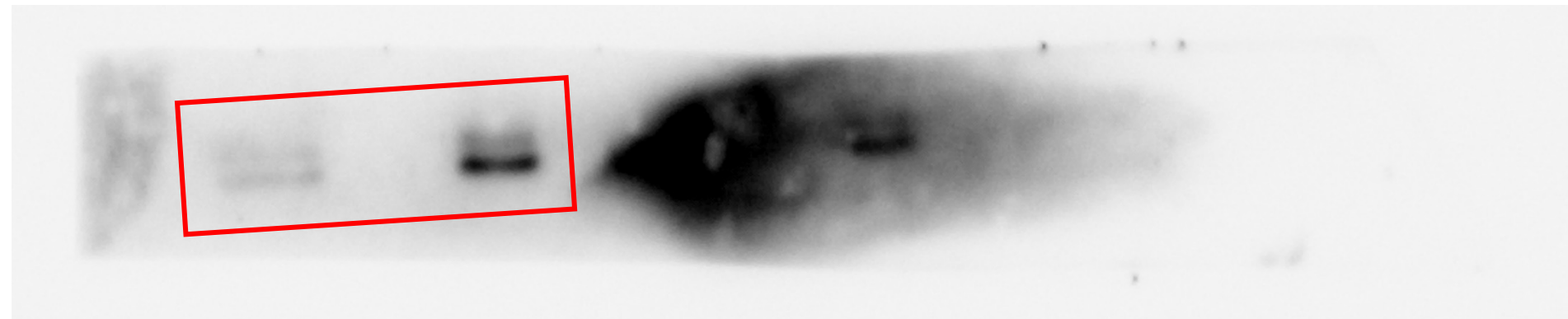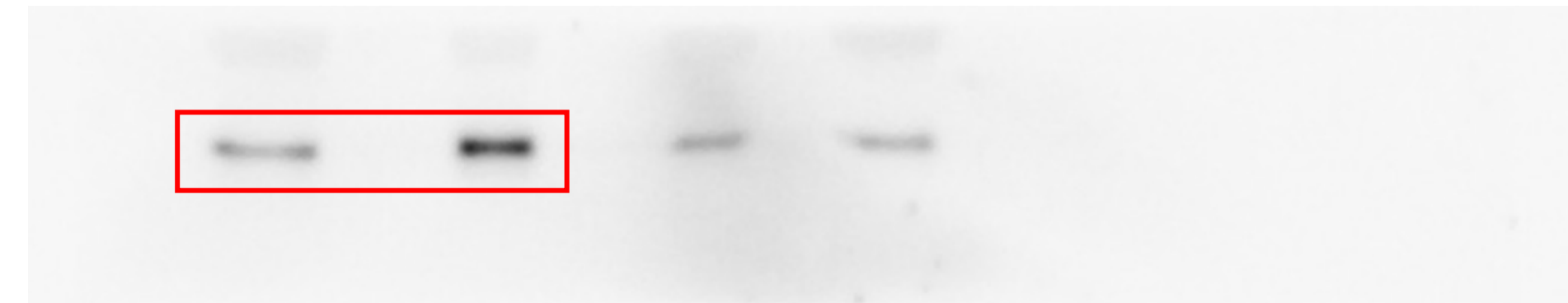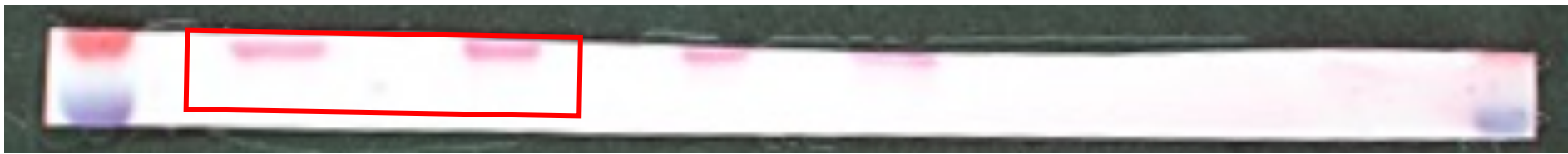

Supplement: Supplementary file 3 — Source Data Fig. 1 [file 44321_2023_13_MOESM3_ESM.zip › EMM-2023-18237_SourceDataForFigure1/1H/western-cropped-areas-1H.pdf]

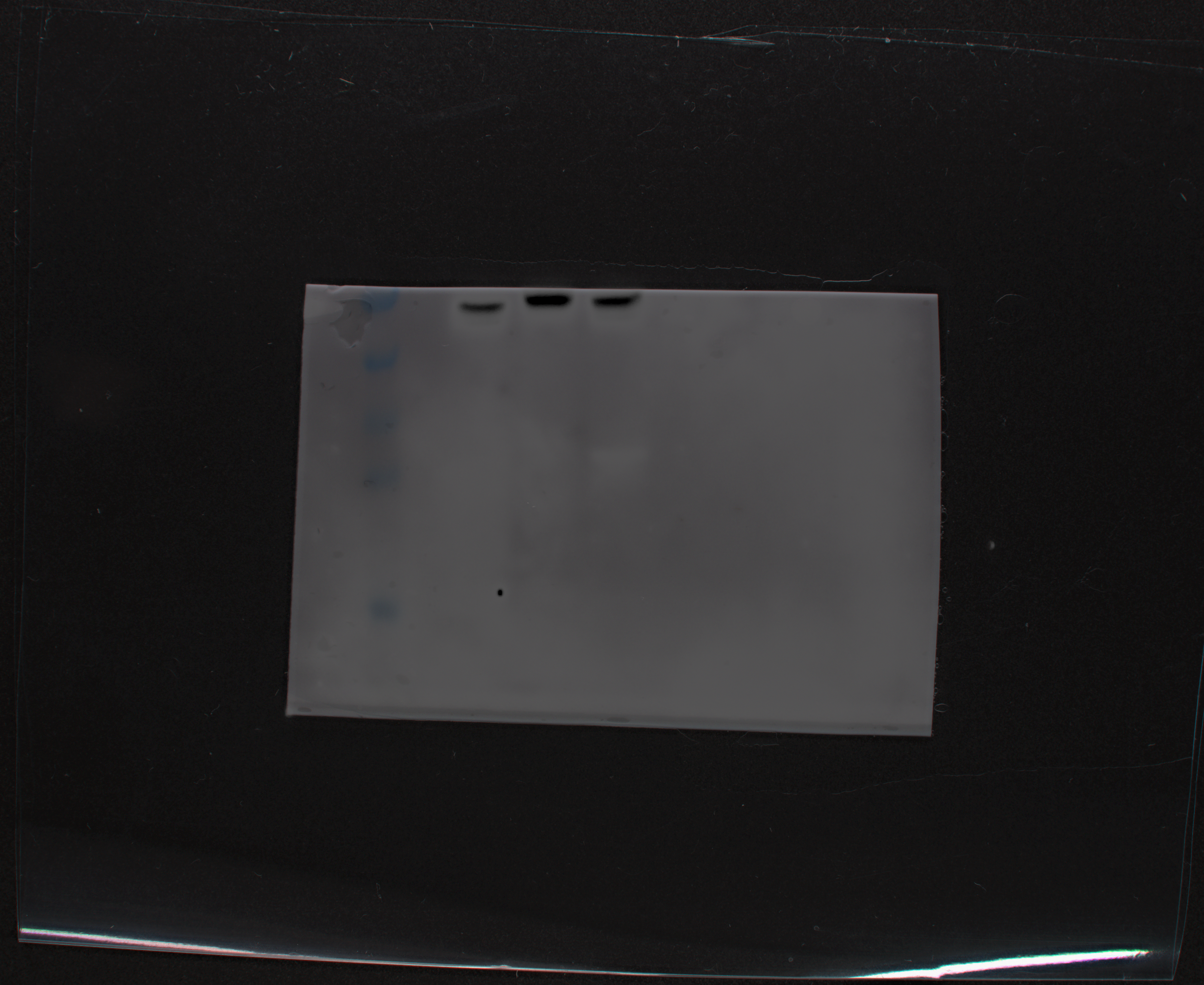

Supplement: Supplementary file 4 — Source Data Fig. 2 [file 44321_2023_13_MOESM4_ESM.zip › EMM-2023-18237_SourceDataForFigure2/2D/western-y-tubulin.tiff]

medium  
GM-CSF  
GM-CSF + static

Bcl-xL

55

40

35

25

15

medium

GM-CSF

GM-CSF + static

$\gamma$ -tubulin

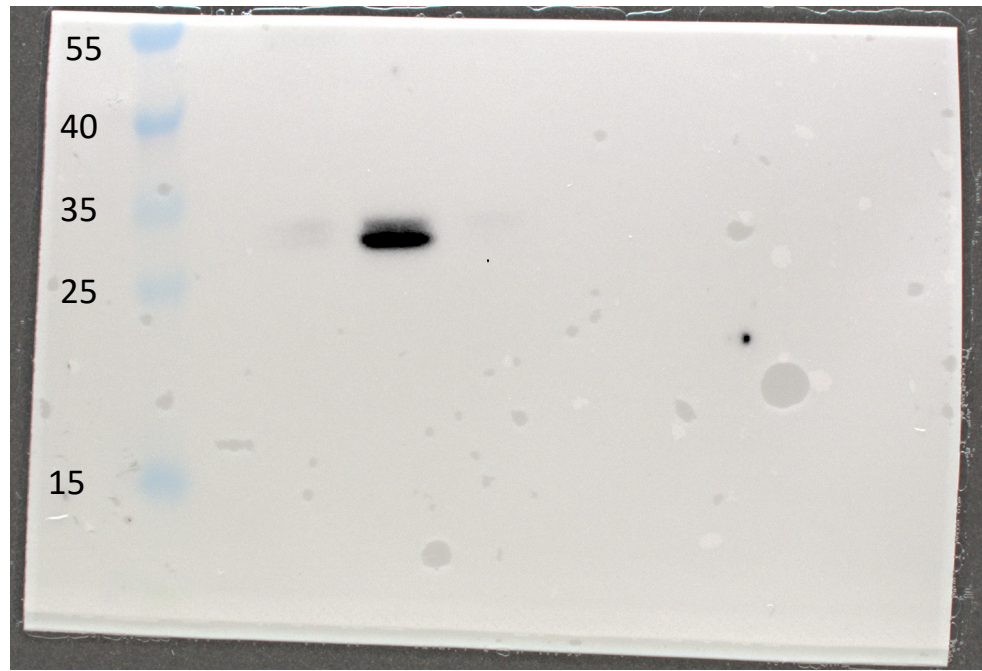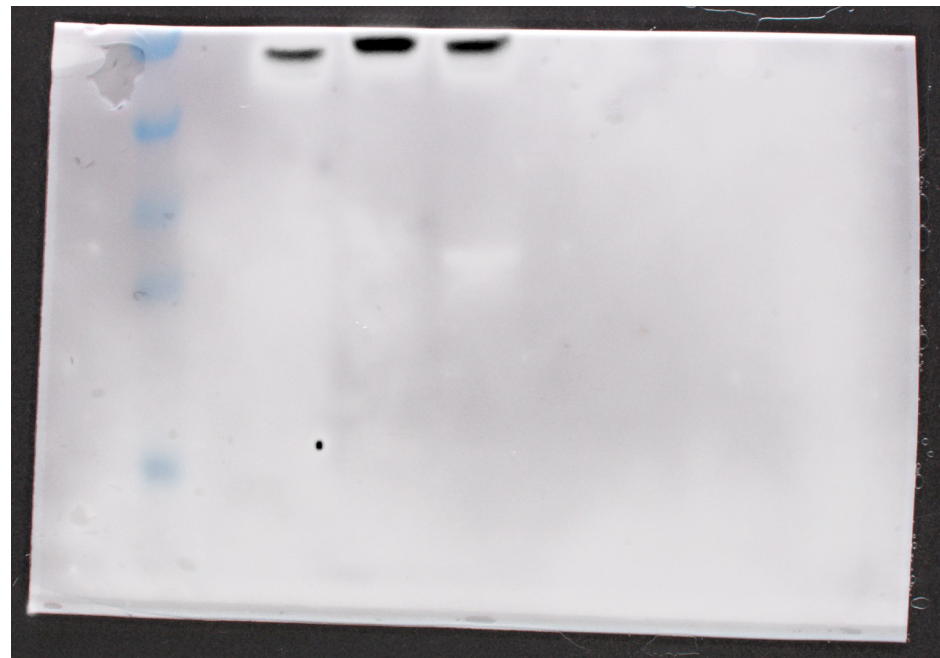

Supplement: Supplementary file 4 — Source Data Fig. 2 [file 44321_2023_13_MOESM4_ESM.zip › EMM-2023-18237_SourceDataForFigure2/2D/Cropped-areas-2D.pdf]

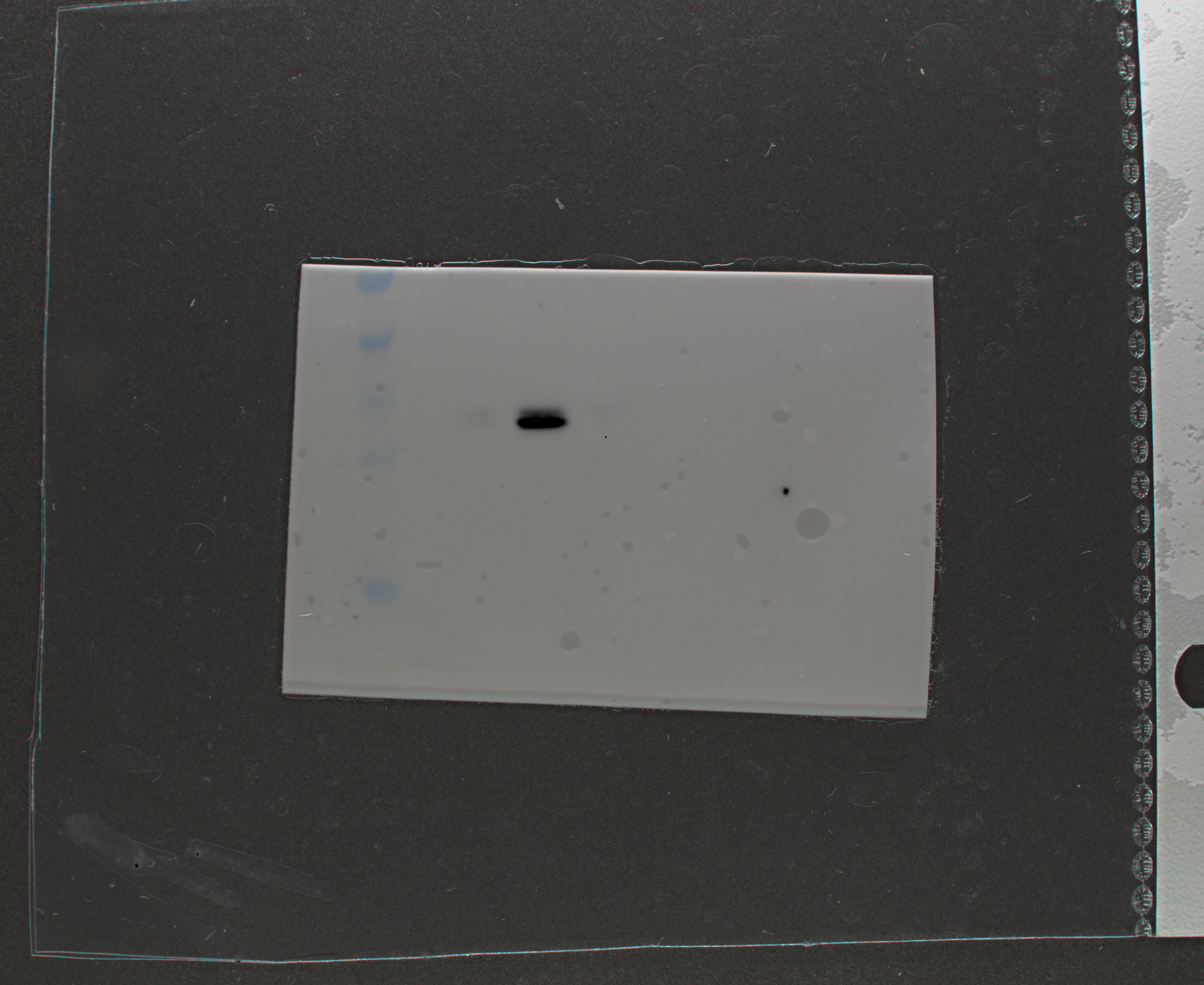

Supplement: Supplementary file 4 — Source Data Fig. 2 [file 44321_2023_13_MOESM4_ESM.zip › EMM-2023-18237_SourceDataForFigure2/2D/western-Bcl-xL-with-scale.tiff]
