# Supplementary material for: Bcl-xL targeting eliminates ageing tumor-promoting neutrophils and inhibits lung tumor growth
Source: EMBO Mol Med. 2023 Dec 20;16(1):10. doi: 10.1038/s44321-023-00013-x (PMC10897164; doi:10.1038/s44321-023-00013-x)
Supplement: Supplementary file 9 — Expanded View Figures [file 44321_2023_13_MOESM9_ESM.pdf]

Expanded View Figures

Figure EV1. Bcl-xL expression analysis in neutrophils and TANs.

(**A**) Gene set enrichment analysis (GSEA) showing downregulation of the apoptosis pathway in TANs compared to HLNs. (**B**) Volcano plot showing differentially expressed genes (DE) in TANs versus HLNs. The anti-apoptotic genes *Bcl2l1*, *Bcl2a1b* and *Mcl1* are highlighted in dark blue. (**C**) Real-time PCR showing *Bcl-2, Bcl2l1, Bcl2a1* and *Mcl1* gene expression in TANs ($n = 11$ biological replicates) normalized to expression in HLNs ($n = 5$). *Rpl30* was used as a reference gene. (**D**) Immunofluorescence staining of neutrophils (MPO) and Bcl-xL in tumors of KP mice. Scale bar: 100 μm. (**E**) Representation of neutrophil subsets in naive and tumor-bearing mice from the available single-cell transcriptomics. *SiglecF*, *Bcl2l1*, *Bcl-2*, *Bcl2a1b* and *Mcl1* expressions are highlighted in green. (**F**) Representative flow cytometry gating strategy of alive bone marrow-extracted neutrophils after 24 h incubation with medium or SV2 SN. Data information: For (**A**), statistical significance was calculated by permutation tests (number of random permutations = 10^5). For (**B**), differential gene expression was computed with limma and significance assessed with the moderated *t* test. Genes with *P* value < 0.01 are highlighted in red ($n = 1335$, $n = 471$ with LFC > 0, $n = 864$ with LFC < 0). Total number of genes tested $n = 5397$. (**C**) Data are shown as mean ± SD and significance was obtained with two-way ANOVA with Sidàk's multiple comparisons test. ns non-significant.

 © The Author(s)

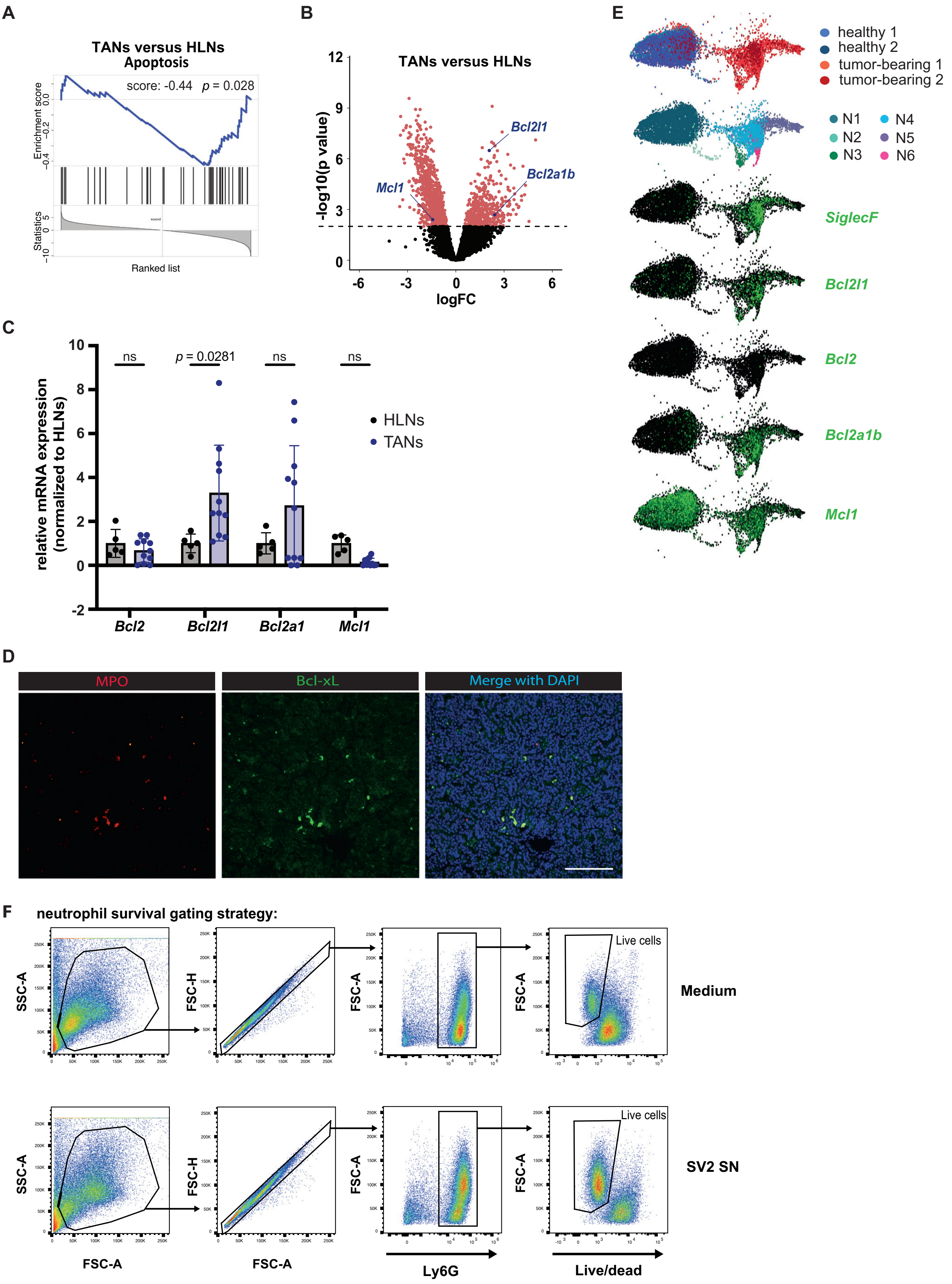
A
TANs versus HLNs
Apoptosis
score: -0.44 p = 0.028
Enrichment score
Statistics
Ranked list
B
TANs versus HLNs
-log10(p value)
logFC
Bcl2l1
Bcl2a1b
Mcl1
C
relative mRNA expression (normalized to HLNs)
ns
p = 0.0281
ns
ns
HLNs
TANs
Bcl2
Bcl2l1
Bcl2a1
Mcl1
D
MPO
Bcl-xL
Merge with DAPI
E
healthy 1
healthy 2
tumor-bearing 1
tumor-bearing 2
N1
N2
N3
N4
N5
N6
SiglecF
Bcl2l1
Bcl2
Bcl2a1b
Mcl1
F
neutrophil survival gating strategy:
SSC-A
FSC-H
FSC-A
Live cells
Medium
SV2 SN
FSC-A
FSC-A
Ly6G
Live/dead

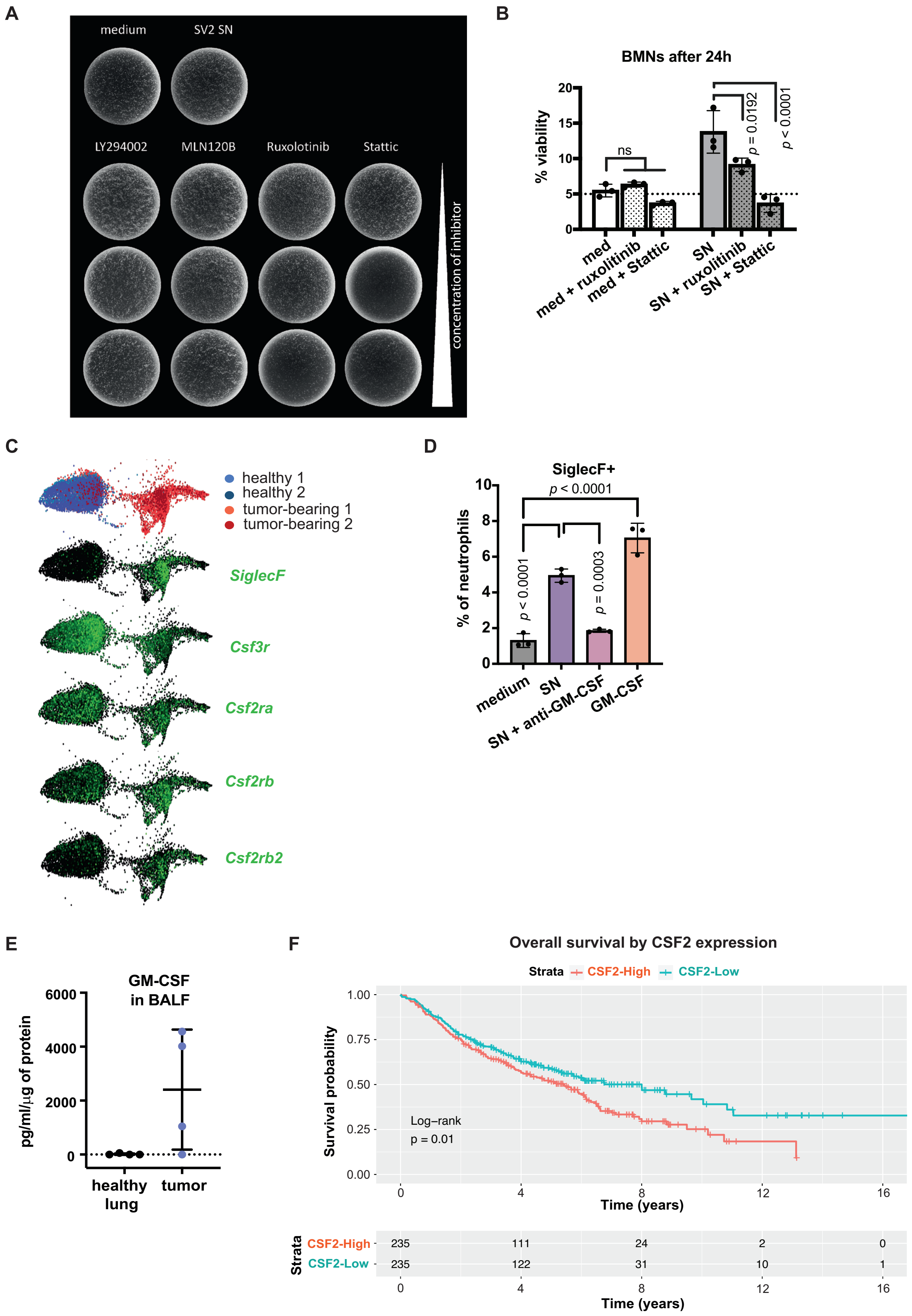
A
medium
SV2 SN
LY294002
MLN120B
Ruxolotinib
Stattic
concentration of inhibitor
B
BMNs after 24h
% viability
ns
p = 0.0192
p < 0.0001
med
med + ruxolitinib
med + Stattic
SN
SN + ruxolitinib
SN + Stattic
C
healthy 1
healthy 2
tumor-bearing 1
tumor-bearing 2
SiglecF
Csf3r
Csf2ra
Csf2rb
Csf2rb2
D
SiglecF+
% of neutrophils
p < 0.0001
p < 0.0001
p = 0.0003
medium
SN
SN + anti-GM-CSF
GM-CSF
E
GM-CSF in BALF
pg/ml/μg of protein
healthy lung
tumor
F
Overall survival by CSF2 expression
Strata
CSF2-High
CSF2-Low
Survival probability
Log−rank
p = 0.01
Time (years)
CSF2-High 235 111 24 2 0
CSF2-Low 235 122 31 10 1

 © The Author(s)

Figure EV2. Bcl-xL induction by GM-CSF-mediated JAK-STAT signaling.

(**A**) Representative images of BMNs incubated with increasing doses of pathway inhibitors observed with brightfield microscopy. (**B**) BMN viability was measured by flow cytometry 24 h after incubation with indicated doses of ruxolitinib (1 μM) or stattic (10 μM) in medium or SV2 supernatant, with BMNs extracted from $n = 3$ mice. (**C**) Representative images of *SiglecF*, *Csf3r*, *Csf2ra*, *Csf2rb* and *Csf2rb2* from publicly available single-cell RNA sequencing data. (**D**) Percentage of $SiglecF^+$ BMNs after 24 h of incubation. $n = 3$ biological replicates. (**E**) GM-CSF concentration measured in the bronchoalveolar lavage fluid (BALF) from healthy ($n = 4$) and tumor-bearing mice ($n = 4$). (**F**) Kaplan–Meier curves for overall survival and *P* value of pairwise differences between groups with high or low *CSF2* expression from the combined LUAD transcriptome dataset. Data information: For (**B**, **D**, **E**), data are shown as mean ± SD. For (**B**, **D**), significance was determined by ordinary one-way ANOVA with Tukey's multiple comparisons test. ns non-significant.

© The Author(s)

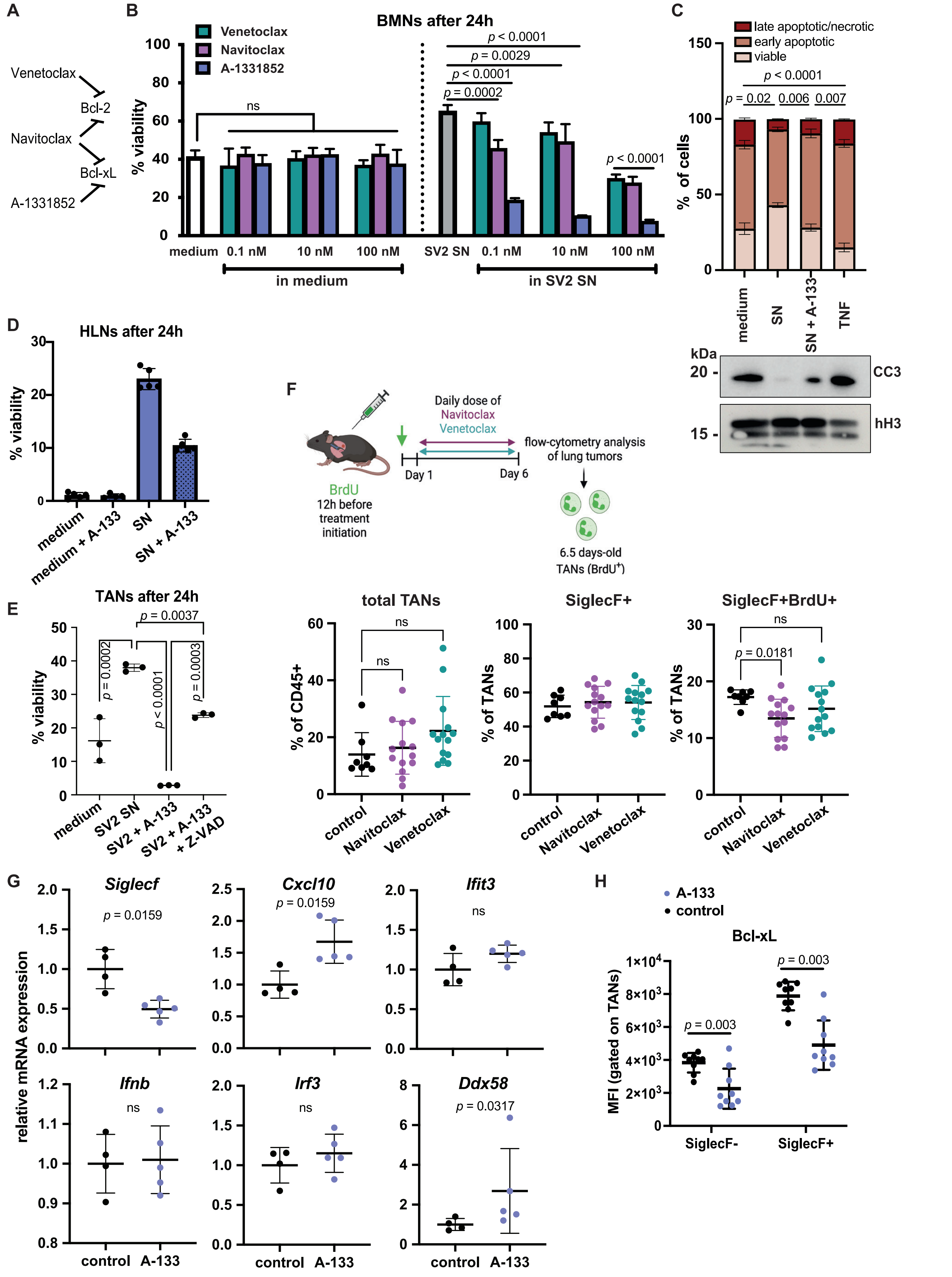
A
Venetoclax
Bcl-2
Navitoclax
Bcl-xL
A-1331852
B
BMNs after 24h
Venetoclax
Navitoclax
A-1331852
% viability
ns
p < 0.0001
p = 0.0029
p < 0.0001
p = 0.0002
p < 0.0001
medium
0.1 nM
10 nM
100 nM
SV2 SN
in medium
in SV2 SN
C
late apoptotic/necrotic
early apoptotic
viable
p < 0.0001
p = 0.02
0.006
0.007
% of cells
medium
SN
SN + A-133
TNF
kDa
CC3
hH3
D
HLNs after 24h
% viability
medium
medium + A-133
SN
SN + A-133
F
Daily dose of
Navitoclax
Venetoclax
flow-cytometry analysis
of lung tumors
Day 1
Day 6
BrdU
12h before
treatment
initiation
6.5 days-old
TANs (BrdU+)
E
TANs after 24h
p = 0.0037
p = 0.0002
p < 0.0001
p = 0.0003
% viability
medium
SV2 SN
SV2 + A-133
SV2 + A-133 + Z-VAD
total TANs
% of CD45+
SiglecF+
% of TANs
SiglecF+BrdU+
p = 0.0181
control
Navitoclax
Venetoclax
G
relative mRNA expression
Siglecf
p = 0.0159
Cxcl10
p = 0.0159
Ifit3
Ifnb
Irf3
Ddx58
p = 0.0317
control
A-133
H
A-133
control
Bcl-xL
MFI (gated on TANs)
p = 0.003
p = 0.003
SiglecF-
SiglecF+

 © The Author(s)

Figure EV3. A-1331852 decreases TAN ageing.

(**A**) Scheme representing BH3-mimetics inhibition specificity. (**B**) Viability (%) of BMNs incubated with 0.1, 10 and 100 nM of Venetoclax, Navitoclax or A-1331852 in medium or SV2 SN for 24 h. $n = 3$ biological replicates. (**C**) Upper part: percentage of viable (AnnexinV$^-$7-AAD$^-$), early (AnnexinV$^+$7-AAD$^-$), and late apoptotic (AnnexinV$^+$7-AAD$^+$) BMNs incubated with medium or SV2 SN with or without A-1331852. TNF (5 ng/mL) was used as control to induce neutrophil apoptosis. $n = 3$ biological replicates. Lower part: Western blot analysis of cleaved-caspase-3 (CC3). Histone H3 (hH3) was used as loading control. (**D**) Healthy lung neutrophils (HLN) survival after 24 h with SV2 SN and with A-1331852 (10 nM) (HLNs were extracted from $n = 5$ healthy non-tumor-bearing mice). (**E**) % of surviving TANs after 24 h, with SV2, SV2 + A-1331852 with or without preliminary incubation with the pan-caspase inhibitor z-VAD-FMK (20 μM). TANs are from $n = 3$ tumors. (**F**) Scheme showing the experimental design and plots showing flow cytometry analysis of neutrophils, SiglecF$^+$ and SiglecF$^+$BrdU$^+$ 6-days-old TANs in control KP mice ($n = 7$ tumors), mice treated with Navitoclax ($n = 12$ tumors) or with Venetoclax ($n = 14$). (**G**) Real-time PCR analysis of expression of the indicated genes in TANs extracted from $n = 4$ control or $n = 5$ A-1331852 treated tumors. (**H**) MFI of Bcl-xL expression in SiglecF$^-$ and SiglecF$^+$ TANs in mice from the same experiment as reported in Fig. 3E. $n = 9$ tumors for each group. Data information: All data are shown as mean ± SD. For (**B**), conditions with drugs in the medium were analyzed compared to the medium-only condition, and drugs in SV2 SN were compared to the SV2 SN condition and significance was determined by ordinary one-way ANOVA with Dunnett's multiple comparisons test. For (**C**), significance was based on two-way ANOVA with Tukey's multiple comparisons test. For (**E**), significance was determined by ordinary one-way ANOVA with Tukey's multiple comparisons test. For (**F**), total TANs were analyzed by Kruskal–Wallis with Dunn's multiple comparisons test. SiglecF$^+$BrdU$^+$ TANs were analyzed by ordinary one-way ANOVA and Dunn's multiple comparisons test. For (**G**), significance was based on a two-tailed Student's *t* test. For (**H**), significance was based on two-way ANOVA. ns non-significant.

© The Author(s)

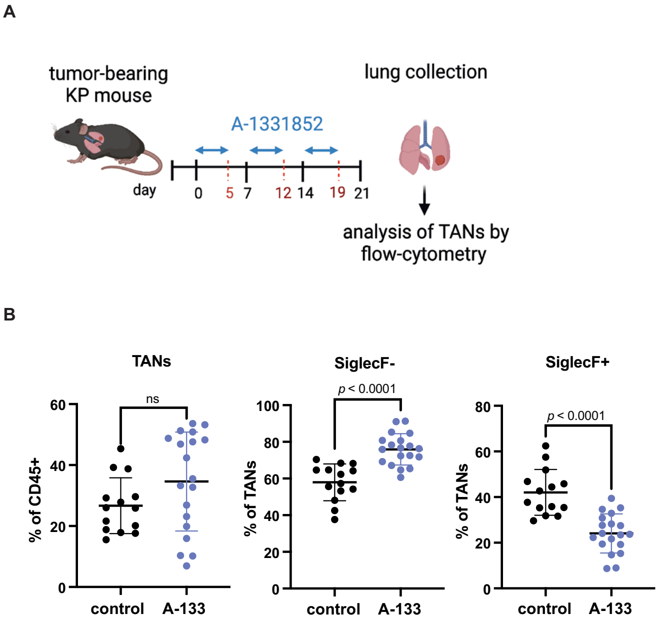

Figure EV4. Intermittent A-1331852 treatment restores selective targeting against SiglecF$^+$ TANs.

(**A**) Scheme describing the treatment regimen and experimental setup. Mice were treated with A-1331852 for 5 days then with two days break, for a duration of 3 weeks. Lung tumors were then isolated and the TAN population was analyzed by flow cytometry. (**B**) Graphs showing the percentages of total TANs, SiglecF$^-$ and SiglecF$^+$ TANs. $n = 14$ tumors were analyzed for control mice (vehicle treated) and $n = 19$ tumors from A-133-treated mice. Data shown are mean ± SD. Significance was determined with a Mann–Whitney test for total TANs and two-tailed t test for SiglecF$^-$ and SiglecF$^+$ percentages. ns non-significant.

 © The Author(s)

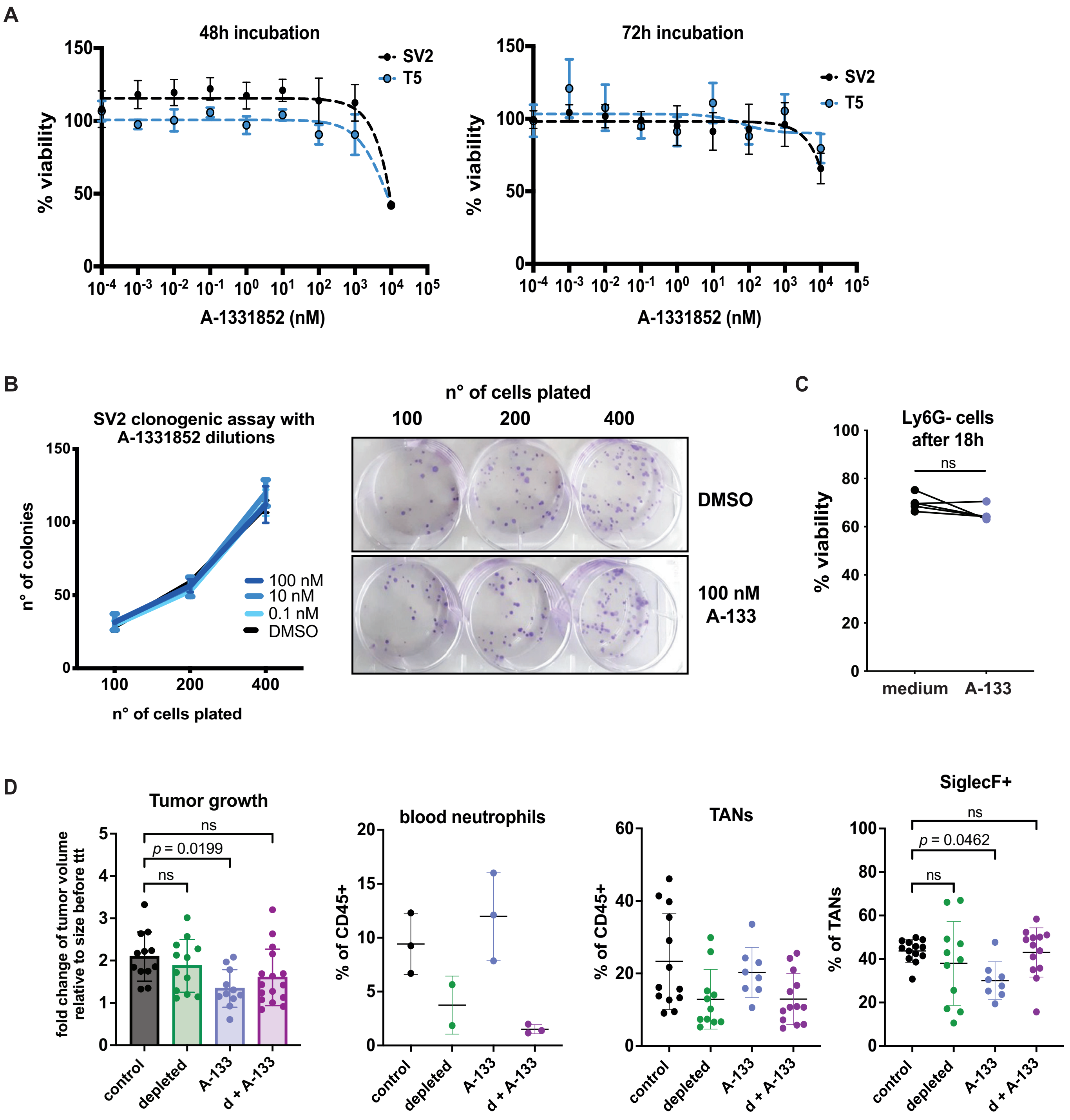

Figure EV5. Bcl-xL blockade does not affect the viability of lung tumor cells.

(**A**) Viability of SV2 and T5 cell lines, measured with PrestoBlue after 48 and 72 h of incubation with serial dilutions of A-1331852. $n = 3$ technical replicates. (**B**) Clonogenic assay performed with 100, 200, 400 single SV2 cells incubated with 0.1, 10 or 100 nM of A-1331852. $n = 3$ technical replicates. (**C**) Data show percentage of viable Ly6G- cells after 18 h of 10 nM of A-1331852 incubation ($n = 5$ tumors). (**D**) Plots showing the growth of single tumors in control ($n = 12$), A-1331852-treated ($n = 11$), neutrophil-depleted ($n = 12$) and neutrophil-depleted in combination with A-1331852 (d + A-133, $n = 16$) KP mice. TAN proportions out of total CD45^{+} and SiglecF^{+} cells out of total TANs are shown for single tumors for control ($n = 13$), A-1331852-treated ($n = 8$), neutrophil-depleted ($n = 10$) and neutrophil-depleted in combination with A-1331852 ($n = 13$). Blood neutrophil levels are also shown for $n = 3$ in control mice, A-1331852-treated and depletion with A-1331852, and $n = 2$ for neutrophil-depleted only mice. Data information: Data are shown as mean ± SD. For (**C**), significance was determined based on a paired t test. For (**D**), ordinary one-way ANOVA with Tukey's multiple comparisons test was performed for the tumor growth and Kruskal–Wallis with Dunn's multiple comparisons test was performed for the other panels. ns, non-significant.
